# Supplementary material for: Persistent activation of monocytes/macrophages and cell senescence in SIV-infected macaques on ART
Source: Front Immunol. 2026 Mar 12;17:1788994. doi: 10.3389/fimmu.2026.1788994 (PMC13018149; doi:10.3389/fimmu.2026.1788994)
Supplement: Supplementary file 1 [file DataSheet1.pdf]

Supplementary Material

Persistent Activation of Monocytes/Macrophages and Cell Senescence in SIV-Infected Macaques on ART

Yilin Chen[1,2], Ding Xiaofeng[1,2], Sonalika Ray[3], Siva Thirugnanam[1], Robert Blair[1], Ahmad Saied[1], Sergiy Sukhanov[4], Jay Kolls[4], Woong-Ki Kim[1,2], Patrick Delafontaine[4], Jay Rappaport[1,2], Xuebin Qin[1,2]*, Namita Rout[1,2]*

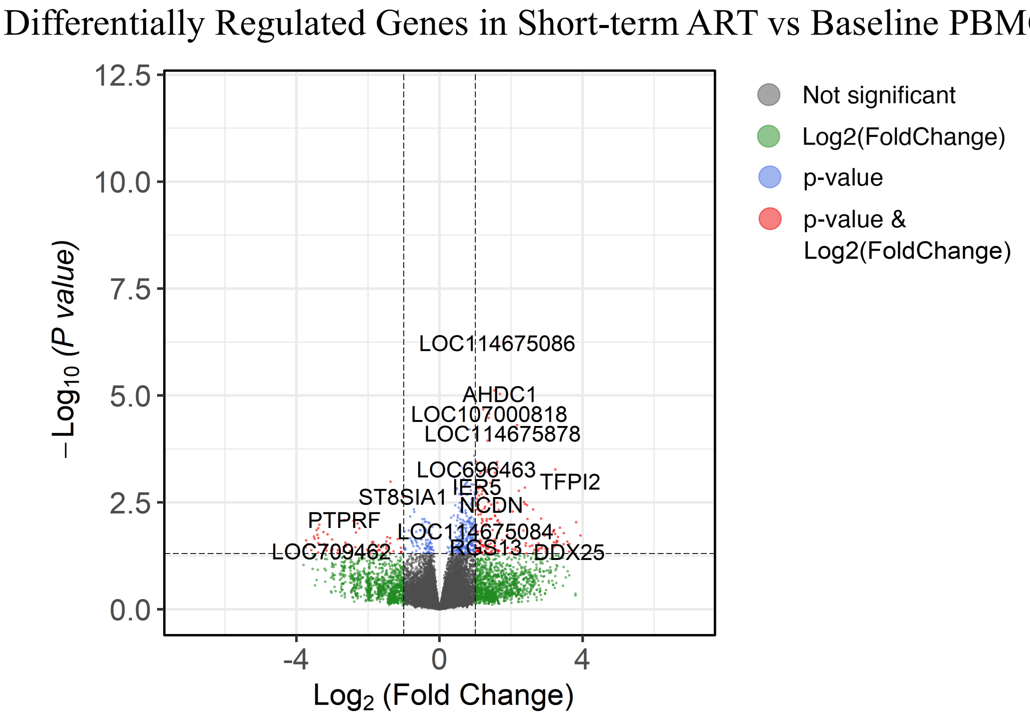

Supplementary Figure 1. Differential gene expression in PBMCs after short-term ART versus baseline. Volcano plot showing differentially expressed genes (DEGs) in SIV-infected + short-term ART vs baseline PBMC.

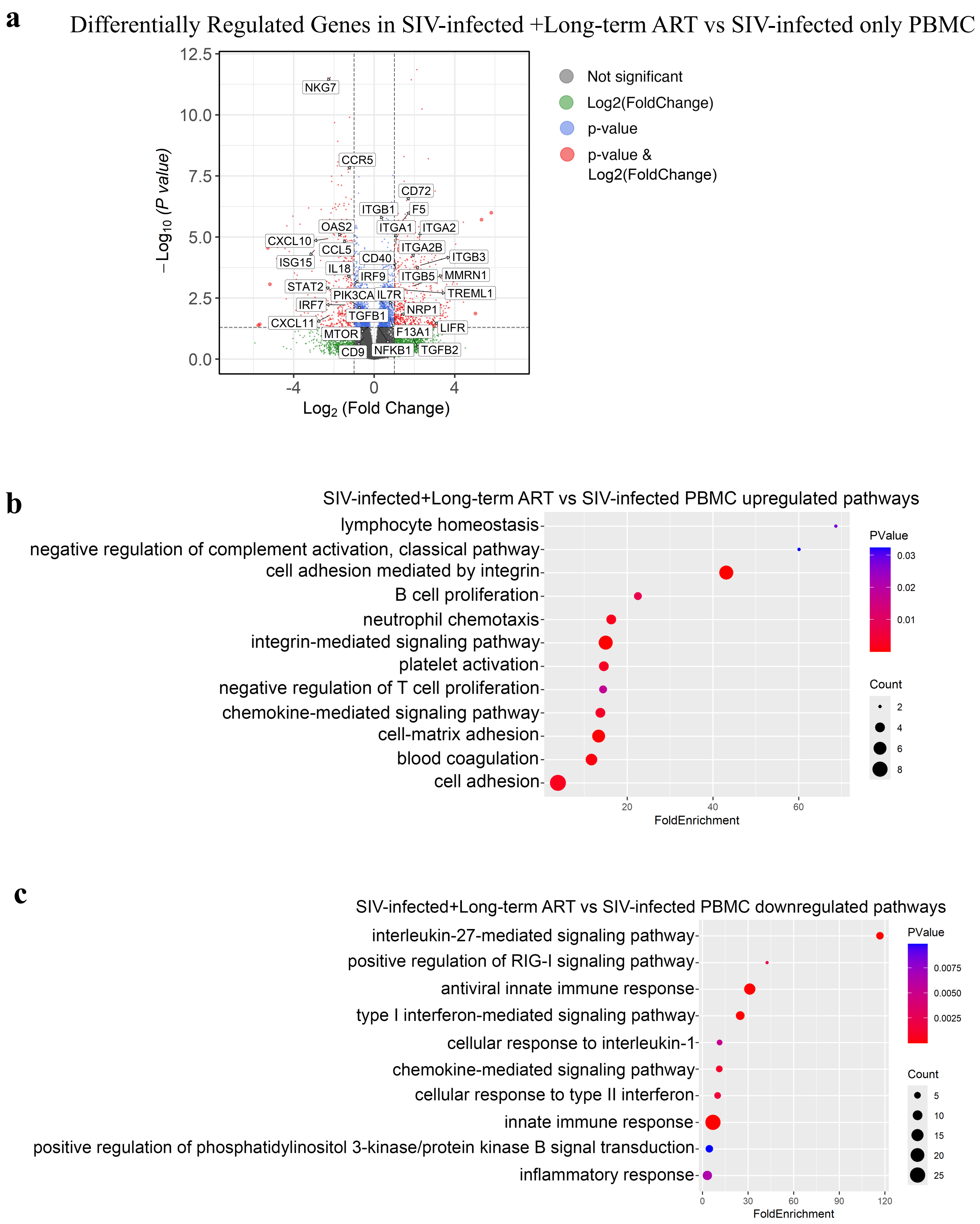

Supplementary Figure 2. Long-term ART treatment continuously downregulates host antiviral innate immune responses but upregulates integrin and coagulation-related signaling pathways.

(a) Volcano plot showing differentially expressed genes (DEGs) in SIV-infected + long-term ART vs SIV-infected macaque PBMC. **(b-c)** Gene Ontology analysis of **(b)** upregulated and **(c)** downregulated DEGs in SIV-infected + long-term ART macaques compared to SIV-infected PBMC.

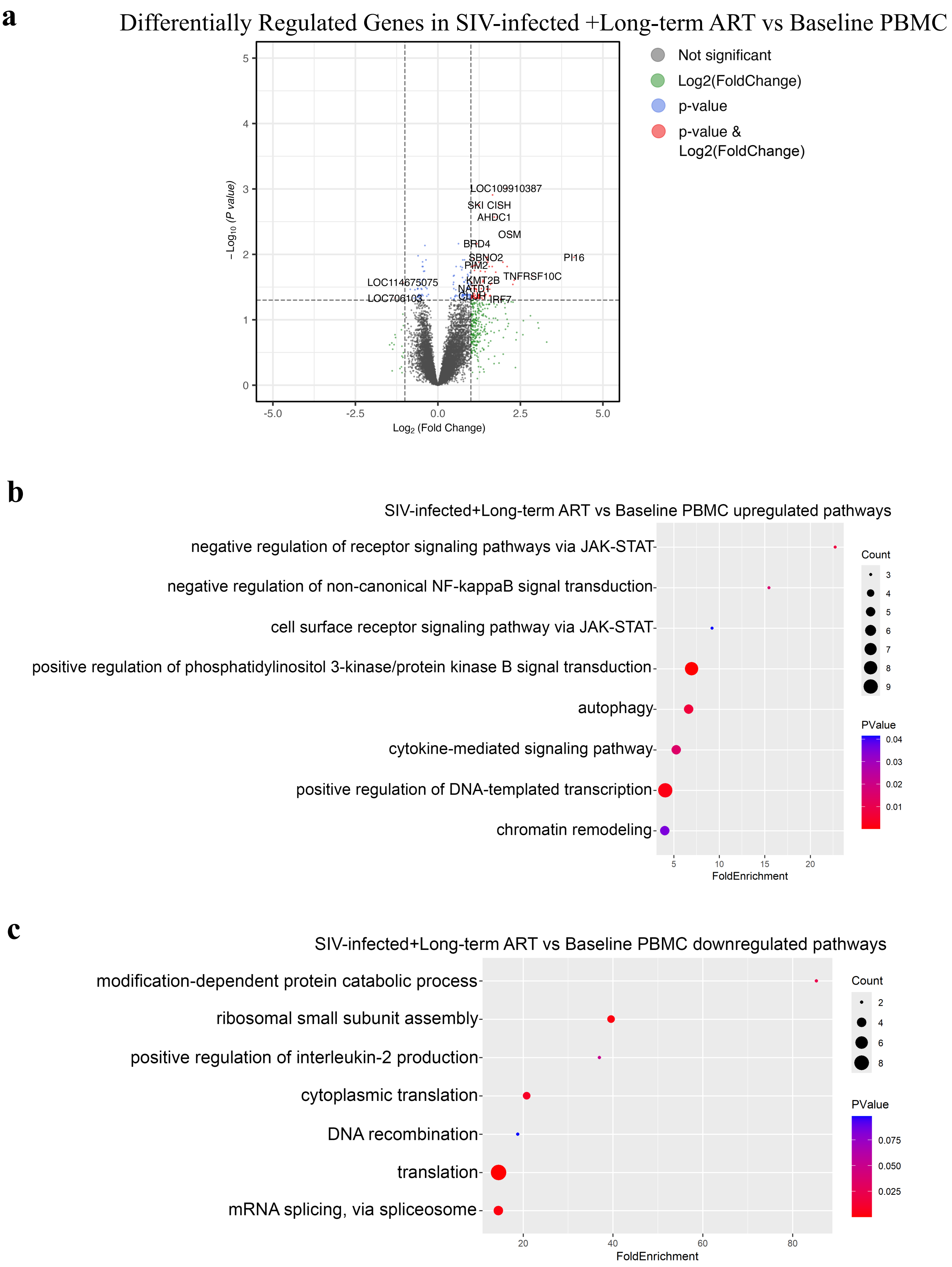

Supplementary Figure 3. Differential expression and pathway enrichment in PBMCs from SIV-infected animals on long-term ART versus baseline. (a) Volcano plot showing differentially

expressed genes (DEGs) in SIV-infected + long-term ART vs baseline monkey PBMC. **(b-c)** Gene Ontology analysis of **(b)** upregulated and **(c)** downregulated DEGs in SIV-infected + long-term ART macaques compared to baseline macaques' PBMC.

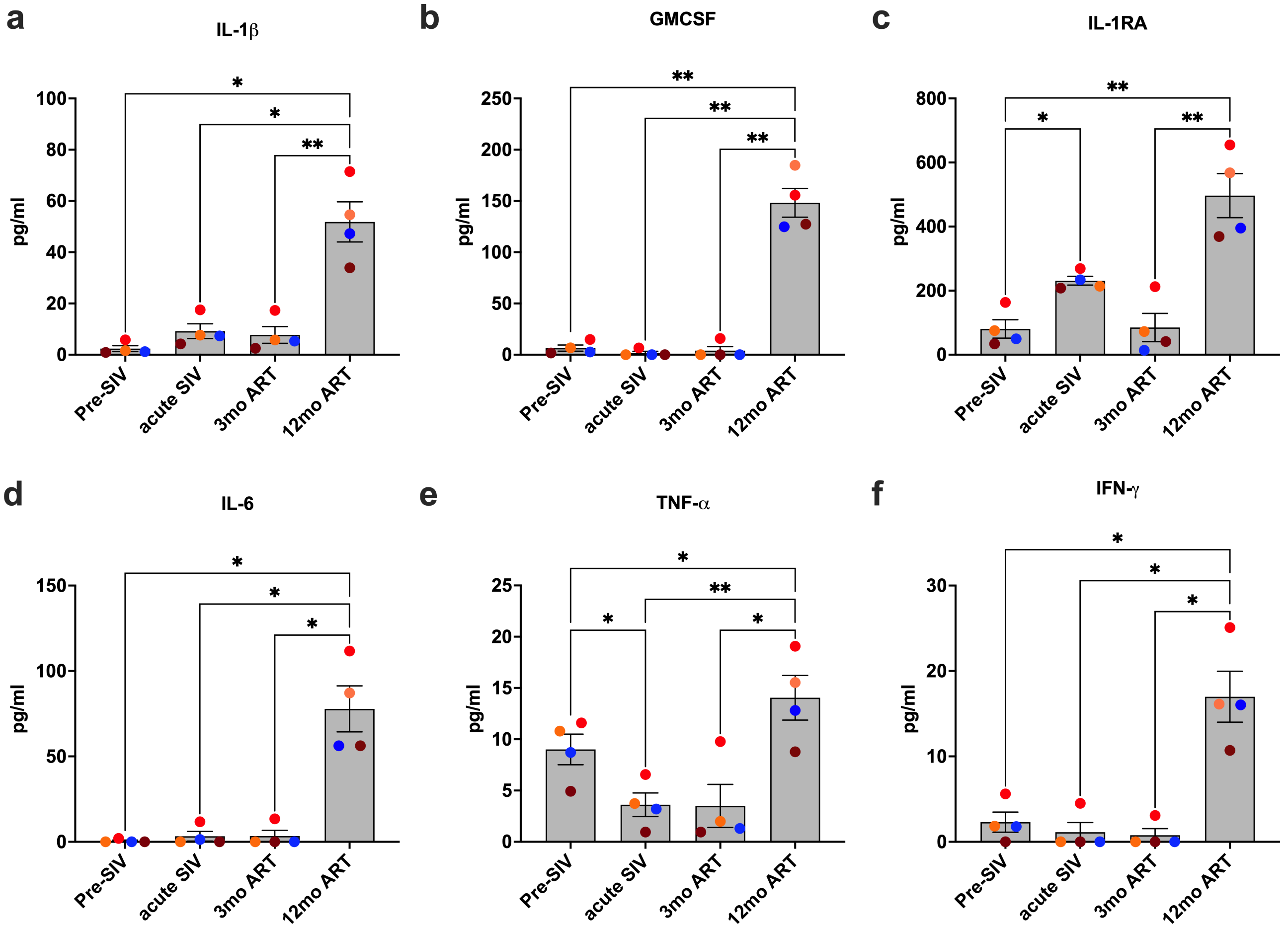

Supplementary Figure 4. Increased pro-inflammatory cytokines in plasma during chronic SIV infection with long-term ART. Multiplex cytokine analysis showing persistently increased plasma levels of inflammatory cytokines through the course of ART-suppressed SIV infection in the 4 SIV-infected rhesus macaques. One-way ANOVA with Tukey's multiple comparisons test was used to determine significant differences between baseline and different time points post-SIV infection and ART. (*$p < 0.05$; **$p < 0.01$; ***$p < 0.001$; ****$p < 0.0001$).

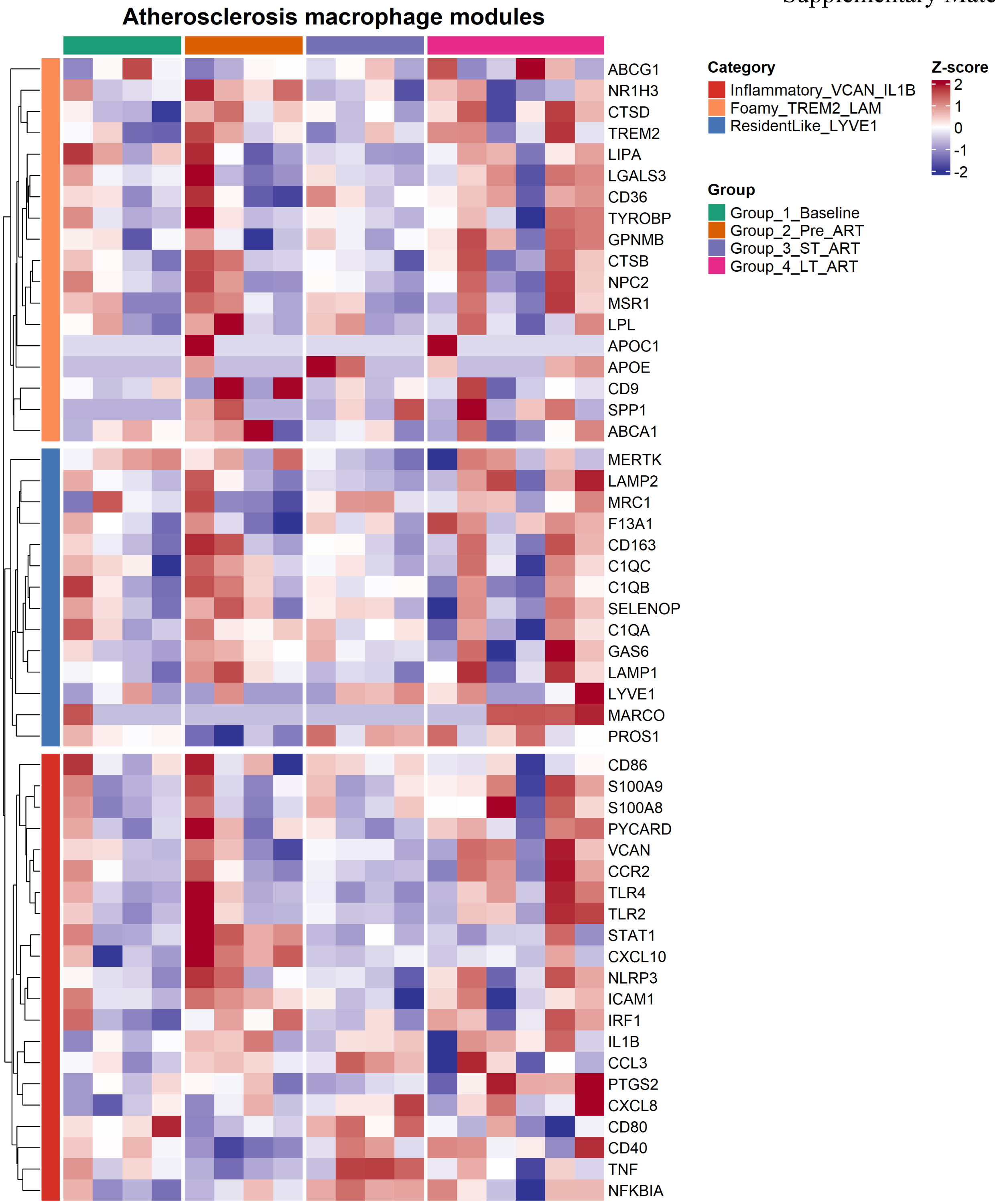

Supplementary Figure 5. Atherosclerosis-associated macrophage modules across SIV and ART states. Heatmap of curated atherosclerosis-associated macrophage signature genes grouped into three: Inflammatory_VCAN/IL1B, Foam_TREM2/LIPA, and Resident_LYVE1 (left color bars).

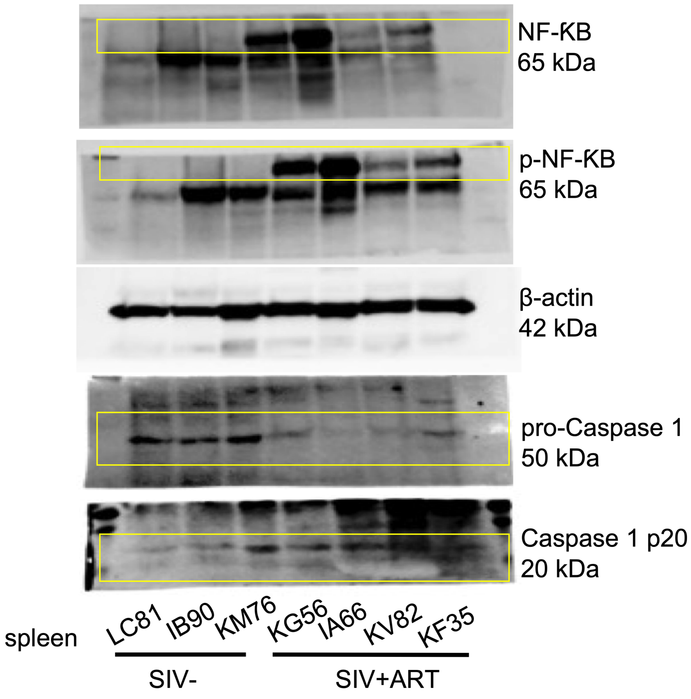

Supplementary Figure 6. The original data for Figure 6a.

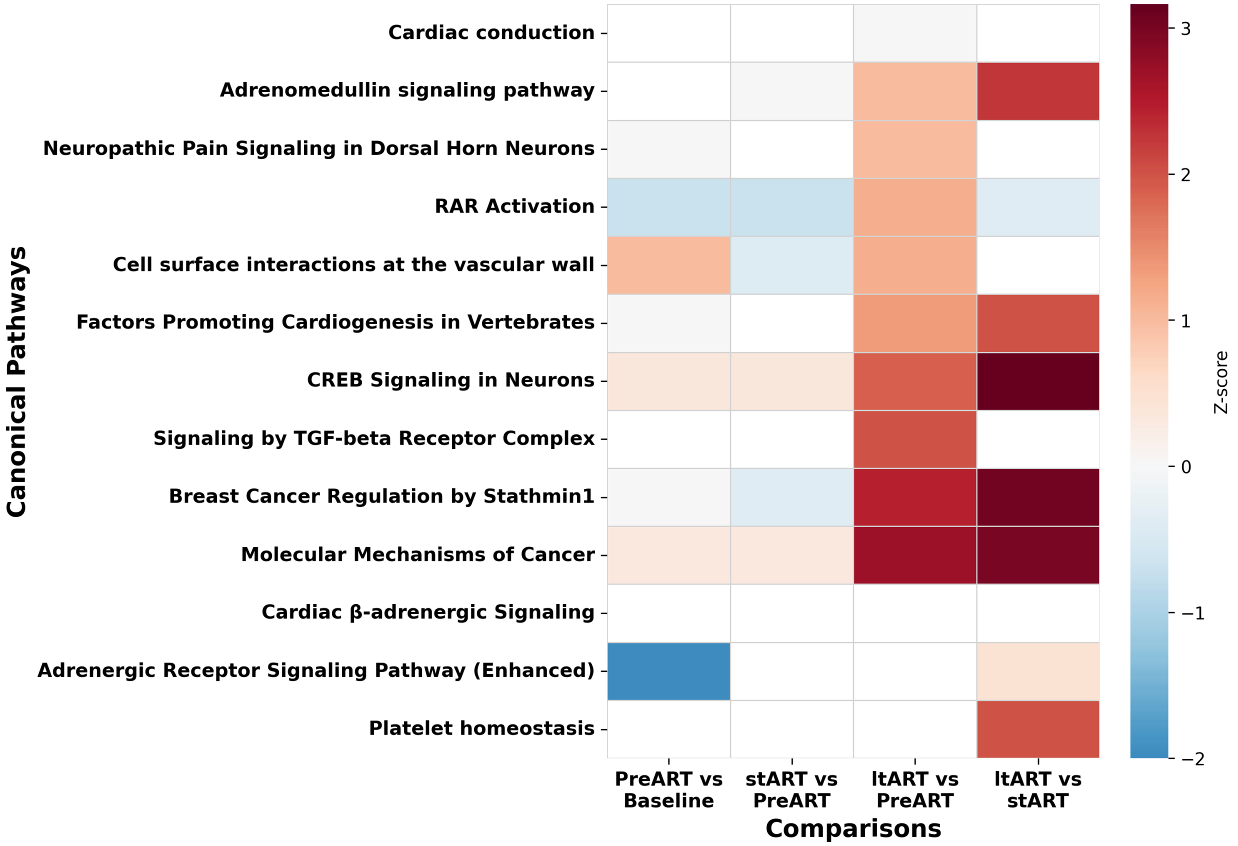

Supplementary Figure 7. CVD-associated pathways and atherogenesis during Chronic SIV+ART. Canonical pathway activity across infection and treatment stages showing persistent activation of cardiovascular signaling pathways during ltART. Heatmap shows predicted activation (red) or inhibition (blue) Z-scores of canonical pathways involved in vascular and cardiac regulation across sequential stages: PreART vs Baseline, short-term ART (stART) vs PreART, long-term ART (ltART) vs PreART, and ltART vs stART. While short-term ART induced limited changes, long-term ART was marked by strong activation of adrenomedullin, β-adrenergic, and platelet homeostasis pathways, as well as vascular interaction and cardiogenic signaling programs. These findings indicate persistent vascular and cardiac stress signaling during chronic infection and prolonged ART.

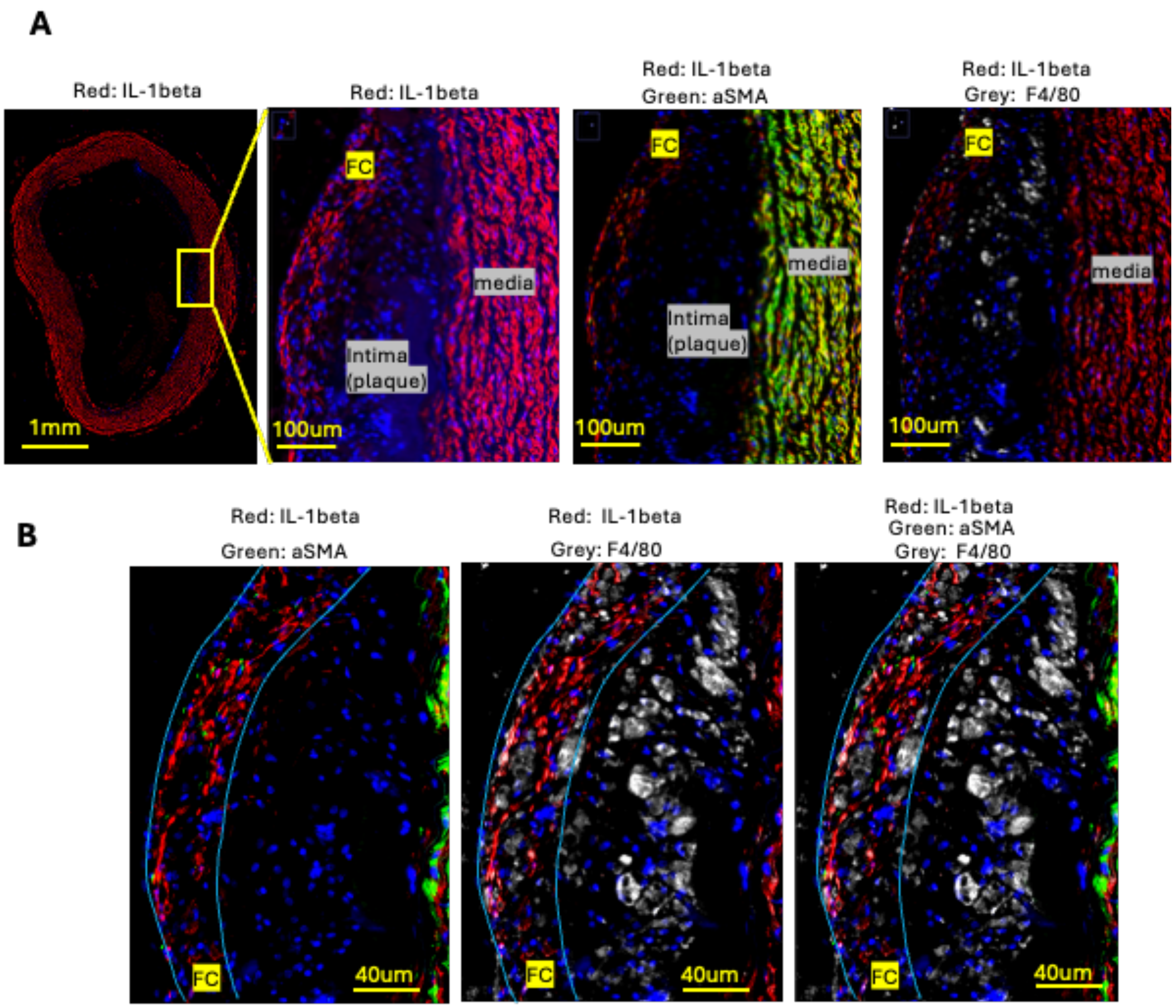

Supplementary Figure 8. IL-1 beta immunopositivity was co-localized with vascular SMC marker in carotid media and with cells in the fibrous cap (FC) in the intima (plaque). A. Il-1beta, aSMA (SMC marker) and F4/80 antigens were co-stained by immunohistochemistry (IHC). Low magnification images show strong IL-1 beta signal in the vascular media and in the fibrous cap. **B,** high magnification images. Il-1 beta signal in FC was co-localized with F4/80-positive macrophages. Blue curves show FC outline. Yellow bar, scale bar.

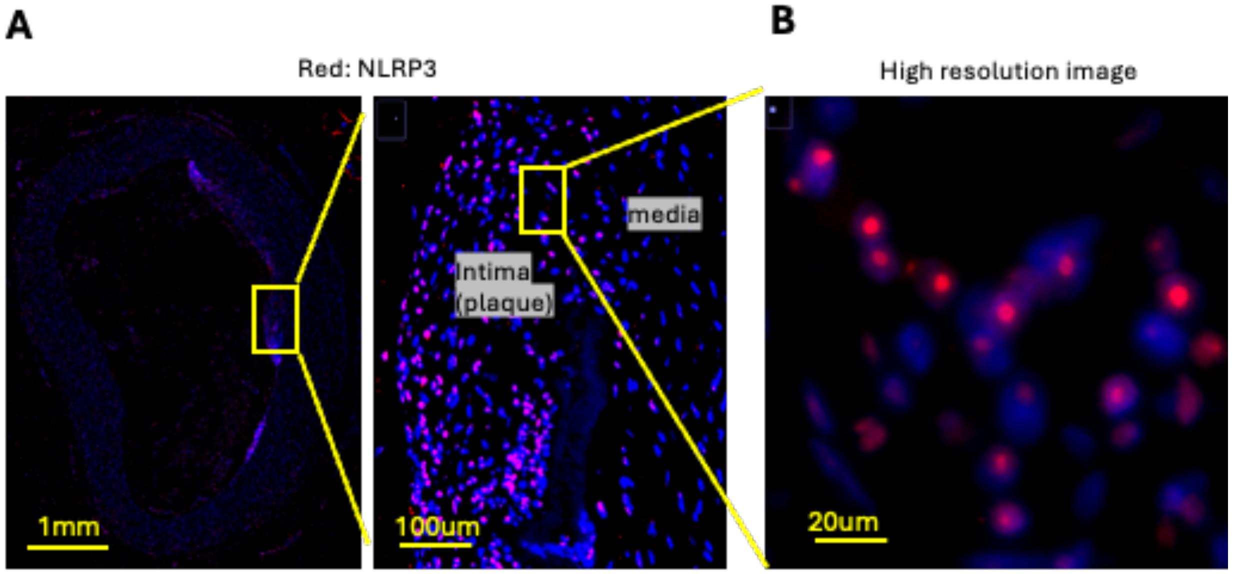

Supplementary Figure 9. NLRP3-immunopositivity in the carotid artery section. A, Carotid artery section was immunostained using NLRP3 antibody and co-stained with DAPI. Low resolution image shows strong NLRP3+ signal detected exclusively in plaque's cells. NLRP3+ signal in media was negligible. **B,** High resolution image show that NLRP3+ signal was mainly nuclear.
