# Supplementary material for: Persistent activation of monocytes/macrophages and cell senescence in SIV-infected macaques on ART
Source: Front Immunol. 2026 Mar 12;17:1788994. doi: 10.3389/fimmu.2026.1788994 (PMC13018149; doi:10.3389/fimmu.2026.1788994)
Supplement: Supplementary file 2 [file DataSheet2.pdf]

Supplemental Tables

Supplementary Table 1: Upregulated Gene Ontology Biological Process Terms in PBMC of Acute SIV-infected monkey as compared to PBMC of uninfected monkeys.

Term	Genes	FoldEnrichment	PValue	FDR
GO:0051607~defense response to virus	PRF1, IFIT5, DDX60L, ADAR, IFIT1, IFI44L, IFIT3, OASL, IFIT2, IFIH1, PYCARD, HERC5, TRIM5, DHX58, ITGAX, TRIM22, ZBP1, BNIP3L, RSAD2, DTX3L, PARP9, UNC13D, PLSCR1, OAS1, OAS2, OAS3, DDIT4, IFIT1B, IRF9, TRIM56, TLR2, NLRC5, IFI6, DDX60, RELA, NLRP3, GBP2, GBP1, GBP3, APOBEC3G, STAT1, TREX1, STAT2, MX2, MX1, EIF2AK2, ISG15, ISG20, MOV10, ZNFX1, MAP3K14, BCL2L1	6.047082002	2.3808E-25	8.61611E-22
GO:0051092~positive regulation of NF-kappaB transcription factor activity	RNF31, SPHK1, EIF2AK2, NOD2, RELA, TRIM8, PYCARD, RPS6KA4, SLCO3A1, TRIM5, TRIM14, NLRP3, TRIM25, PRKD2, RBCK1, ZBTB7A, TLR4, TRIM22, CARD11, TLR2	4.670983213	4.78563E-08	1.44327E-05
GO:0070106~interleukin-27-mediated signaling pathway	OAS1, STAT1, OAS2, OAS3, MX1, OASL	21.01942446	2.89904E-06	0.000499601
GO:0032729~positive regulation of type II interferon production	SASH3, SLC11A1, ARID5A, ISG15, TYK2, CD3E, CD2, PYCARD, KLRK1, ABL1, SLAMF6, TLR4, IL12RB2	4.670983213	1.91667E-05	0.002477292
GO:0060337~type I interferon-mediated signaling pathway	IFIH1, HDAC4, OAS1, STAT1, OAS2, STAT2, OAS3, TREX1, TYK2, OASL	5.605179856	5.94307E-05	0.006721242
GO:0050852~T cell receptor signaling pathway	RNF31, PIK3CD, THY1, CD3E, FOSL2, ZAP70, THEMIS2, CD8A, INPP5D, ABL1, CSK, PRKD2, PLCG1, RBCK1, TNFRSF21	3.474285035	0.000104516	0.010506775
GO:0032755~positive regulation of interleukin-6 production	SIGLEC16, CD74, TGFB1, STAT3, ARID5A, NOD2, RELA, EREG, IL17RA, IFIH1, PYCARD, TLR4, TLR2	3.57192834	0.000274067	0.02204108
GO:0032728~positive regulation of interferon-beta production	IFIH1, OAS1, OAS2, OAS3, ISG15, TLR4, TRIM56, TLR2	5.214120796	0.000745054	0.042799214
GO:0070374~positive regulation of ERK1 and ERK2 cascade	PDGFRB, CD74, CSF1R, TGFB1, NOTCH1, SHC1, C5AR1, FPR2, ARRB2, NOD2, VEGFA, PYCARD, RAPGEF1, ABL1, PTK2B, PRKD2, PDE8B, TLR4	2.43703472	0.001233681	0.061160175
GO:0000165~MAPK cascade	MAP2K3, MAP2K2, IGFBP4, MINK1, MAPK8IP3, DUSP6, ITPKB, MAPKAPK2, PTK2B, PTPN7, CCR5, MAP3K14, MAP3K11	2.986366317	0.00138287	0.06585007
GO:0043123~positive regulation of canonical NF-kappaB signal transduction	RNF31, CX3CR1, IFIT5, FASLG, NOD2, RELA, TRIM8, PYCARD, CASP10, TRIM5, NUP62, ABL1, TNFSF10, FLNA, TRIM25, TRIM22, BRD4, CD74, TGFB1, STAT3, TNFRSF1A, VEGFA, SLCO3A1, LTBR, RBCK1, TLR4, CARD11	3.26163483	2.56629E-07	6.61073E-05

P value: nominal p value, FDR: False Discovery Rate (Benjamini–Hochberg method)

Supplementary Table 2: Downregulated Gene Ontology Biological Process Terms in PBMC of Acute SIV-infected monkey as compared to PBMC of uninfected monkeys.

Category	Term	Count	%	PValue	Genes	List Total	Pop Hits	Pop Total	FoldEnrichment	Bonferroni	Benjamini	FDR
GOTERM_BP_DIRECT	GO:0042113~B cell activation	6	1.485148515	0.001308805	FCRL1, CR2, BANK1, MS4A1, HDAC9, MALT1	382	42	19478	7.284218399	0.89473861	0.173064232	0.172258813
GOTERM_BP_DIRECT	GO:0042100~B cell proliferation	5	1.237623762	0.003360841	MEF2C, CR2, LEF1, IL7R, MS4A1	382	32	19478	7.967113874	0.996932877	0.385152348	0.3833599
GOTERM_BP_DIRECT	GO:0001780~neutrophil homeostasis	3	0.742574257	0.01867657	PDE4B, GCNT4, JAM3	382	11	19478	13.90623513	1	1	0.995925495
GOTERM_BP_DIRECT	GO:0002456~T cell mediated immunity	3	0.742574257	0.038218289	CR2, CR1, CD46	382	16	19478	9.560536649	1	1	0.995925495
GOTERM_BP_DIRECT	GO:0031295~T cell costimulation	4	0.99009901	0.051561908	DPP4, PIK3CA, CD28, ICOS	382	43	19478	4.743211981	1	1	0.995925495

P value: nominal p value, FDR: False Discovery Rate (Benjamini–Hochberg method)

Supplementary Table 3: Upregulated Gene Ontology Biological Process Terms in PBMC of SIV-infected monkey with short-term ART as compared to PBMC of Acute SIV-infected monkeys.

Category	Term	Genes	FoldEnrichment	PValue	FDR
GOTERM_BP_DIRECT	GO:0042100~B cell proliferation	CD79A, MEF2C, CR2, CD40, CD40LG, LEF1, IL7R, MS4A1	16.56292517	3.95639E-07	0.000114656
GOTERM_BP_DIRECT	GO:0030183~B cell differentiation	ITGB1, CD79B, CD79A, CR2, CD40LG, BLNK, ZBTB1, PAX5, MS4A1, HDAC9	9.331225448	1.14819E-06	0.000277288
GOTERM_BP_DIRECT	GO:0042113~B cell activation	FCRL1, CD79A, CR2, CD40, FCRL5, BANK1, MS4A1, HDAC9	12.61937156	2.78818E-06	0.000577152
GOTERM_BP_DIRECT	GO:0050853~B cell receptor signaling pathway	BLK, CD79B, CD79A, MEF2C, BANK1, BLNK, CTLA4, MS4A1	8.983281448	2.83437E-05	0.004107008
GOTERM_BP_DIRECT	GO:0031295~T cell costimulation	DPP4, CD40LG, CD28, TNFRSF13C, ICOS	7.703686126	0.003902986	0.332672132
GOTERM_BP_DIRECT	GO:0023035~CD40 signaling pathway	ITGB1, CD40, CD40LG	19.8755102	0.009370546	0.646567682
GOTERM_BP_DIRECT	GO:0060390~regulation of SMAD protein signal transduction	TOB1, SPTBN1	66.25170068	0.029859705	0.994509266
GOTERM_BP_DIRECT	GO:0030890~positive regulation of B cell proliferation	MEF2C, CD40, GPR183, TNFRSF13C	5.638442611	0.033598305	0.994509266
GOTERM_BP_DIRECT	GO:0030593~neutrophil chemotaxis	ITGA1, PPBP, PF4V1, PF4	4.491640724	0.059168699	0.994509266

P value: nominal p value, FDR: False Discovery Rate (Benjamini–Hochberg method)

Supplementary Table 4: Downregulated Gene Ontology Biological Process Terms in PBMC of SIV-infected monkey with short-term ART as compared to PBMC of Acute SIV-infected monkeys.

Term	Genes	FoldEnrichment	PValue	FDR
GO:0140374~antiviral innate immune response	ZBP1, MX1, EIF2AK2, IFIT1, IFIT3, IFIT2, OASL, IFIH1, CXCL10, OAS1, OAS2, OAS3, DHX58, TRIM25, NLRP1, TRIM21	9.097883521	1.45474E-10	5.82175E-08
GO:0070106~interleukin-27-mediated signaling pathway	OAS1, STAT1, OAS2, OAS3, MX1, OASL	27.72011385	7.37867E-07	0.00014654
GO:0071346~cellular response to type II interferon	GBP6, DAPK1, STAT1, FCAR, CCL5, ZYX, SIRPA, CALM3, GBP2, GBP1, TLR4, GBP3, TLR2	6.1600253	1.14023E-06	0.000199808
GO:0032755~positive regulation of interleukin-6 production	SIGLEC16, TGFB1, UNC93B1, CARD9, STAT3, PTAFR, NOD2, IL17RA, IFIH1, PYCARD, TYROBP, TLR8, TLR4, TLR2	5.072962012	3.65441E-06	0.000544325
GO:0051092~positive regulation of NF-kappaB transcription factor activity	RNF31, SPHK1, CARD9, EIF2AK2, NOD2, PYCARD, RPS6KA4, SLCO3A1, TRIM5, TRIM14, NLRP3, TRIM25, ZBTB7A, TLR4, TLR2	4.620018975	4.49239E-06	0.000637278
GO:0060337~type I interferon-mediated signaling pathway	IFIH1, HDAC4, IFI27, OAS1, STAT1, OAS2, STAT2, OAS3, TREX1, OASL	7.392030361	6.66671E-06	0.000902733
GO:0032731~positive regulation of interleukin-1 beta production	PYCARD, EGR1, NLRP12, TYROBP, STAT3, NLRP3, TLR8, NLRP1, NOD2, MEFV, TLR4	6.1600253	1.02789E-05	0.001301987
GO:0070374~positive regulation of ERK1 and ERK2 cascade	PDGFRB, CCR1, NOTCH2, CSF1R, TGFB1, NOTCH1, SHC1, CARD9, C5AR1, FPR2, ARRB2, NOD2, PYCARD, GLIPR2, RAPGEF1, PTK2B, SPRY2, PDE8B, TLR4	3.392477702	1.39385E-05	0.001597033
GO:0043123~positive regulation of canonical NF-kappaB signal transduction	RNF31, CX3CR1, TGFB1, CARD9, STAT3, IFIT5, NOD2, TNFRSF1A, PYCARD, SLCO3A1, CASP10, TRIM5, FLNA, TLR8, TRIM25, S100A4, LTBR, TRIM21, TLR4, BRD4	3.186219983	1.89485E-05	0.002020791
GO:0051897~positive regulation of phosphatidylinositol 3-kinase/protein kinase B signal transduction	PDGFRB, CX3CR1, CSF1R, TGFB1, HIP1, GPX1, SEMA4D, FN1, PRR5L, FPR2, ARRB2, THBS1, PIK3R5, FGR, CCL5, PTK2B, SPRY2, EXTL3	3.261189865	4.14204E-05	0.00362916
GO:0032733~positive regulation of interleukin-10 production	PYCARD, SASH3, RBM47, STAT3, ISG15, NOD2, TLR4, TLR2	7.040028915	0.000119569	0.009373542
GO:0030316~osteoclast differentiation	CSF1R, SBNO2, TGFB1, TYROBP, GAB2, TCIRG1, SLC4A2, FOSL2	6.427852487	0.000216128	0.015703534
GO:0032757~positive regulation of interleukin-8 production	PYCARD, FCN1, AFAP1L2, STAT3, TLR8, CD14, NOD2, TLR4, TLR2	5.11755948	0.000350145	0.020861655
GO:0002430~complement receptor mediated signaling pathway	ITGAM, C5AR1, FPR1, FPR3, FPR2	12.3200506	0.000566203	0.031222776
GO:0002221~pattern recognition receptor signaling pathway	PYCARD, NLRP3, NLRP1, NOD2, MEFV	10.87063288	0.000945766	0.046187493
GO:0042098~T cell proliferation	SPN, SASH3, TGFB1, SH2D2A, MSN, TNFSF13B, IDO1	5.88002415	0.001113064	0.051326235
GO:0060340~positive regulation of type I interferon-mediated signaling pathway	ZBP1, RBM47, NLRC5, ADAR, TRIM56	8.800036143	0.002183288	0.079317257
GO:0030225~macrophage differentiation	CSF1R, IL15, CASP10, UCP2, GAB3, FOSL2	6.1600253	0.002659828	0.092135202
GO:0060907~positive regulation of macrophage cytokine production	PYCARD, CARD9, MAPKAPK2, NOD2, TLR4	7.392030361	0.004237611	0.123763177
GO:0002224~toll-like receptor signaling pathway	UNC93B1, MAPKAPK2, TLR8, TLR4, TLR2	6.844472556	0.005634486	0.155417903

P value: nominal p value, FDR: False Discovery Rate (Benjamini–Hochberg method)

Supplementary Table 5: Upregulated Gene Ontology Biological Process Terms in PBMC of SIV-infected monkey with long-term ART as compared to PBMC of Acute SIV-infected monkeys.

Category	Term	Genes	FoldEnrichment	PValue	FDR
GOTERM_BP_DIRECT	GO:0030593~neutrophil chemotaxis	ITGA1, PPBP, PF4V1, PF4	16.30299226	0.001837986	0.133679958
GOTERM_BP_DIRECT	GO:0070098~chemokine-mediated signaling pathway	PPBP, CCR4, PF4V1, PF4	13.74109347	0.00299638	0.151959286
GOTERM_BP_DIRECT	GO:0042100~B cell proliferation	CR2, LEF1, IL7R	22.54398148	0.007628697	0.270818729
GOTERM_BP_DIRECT	GO:0042130~negative regulation of T cell proliferation	CR1, BTLA, PDE5A	14.42814815	0.01796583	0.554597348
GOTERM_BP_DIRECT	GO:0002260~lymphocyte homeostasis	SLC40A1, SKIL	68.70546737	0.028402871	0.695380625
GOTERM_BP_DIRECT	GO:0033627~cell adhesion mediated by integrin	ITGB1, MMRN1, ITGB5, ITGA2, ITGB3, ITGA1, ITGA2B	43.16112694	1.16163E-08	8.24761E-06
GOTERM_BP_DIRECT	GO:0007229~integrin-mediated signaling pathway	ITGB1, NRP1, ITGB5, ITGA2, ITGB3, ITGA1, ITGA2B	15.02932099	6.71549E-06	0.001589333
GOTERM_BP_DIRECT	GO:0007160~cell-matrix adhesion	ITGB1, ITGB5, ITGA2, ITGB3, ITGA1, ITGA2B	13.35939643	8.24786E-05	0.014639954
GOTERM_BP_DIRECT	GO:0007596~blood coagulation	MMRN1, ITGA2, ITGB3, F13A1, F5	11.67325902	0.000856967	0.101407711
GOTERM_BP_DIRECT	GO:0007155~cell adhesion	SELP, ITGB1, MMRN1, ITGB5, ITGA2, ITGB3, ITGA1, ITGA2B, CD9	3.871596104	0.002071098	0.133679958
GOTERM_BP_DIRECT	GO:0030168~platelet activation	TREML1, ITGB3, CD9, PF4	14.57388702	0.002534445	0.13841971

P value: nominal p value, FDR: False Discovery Rate (Benjamini–Hochberg method)

Supplementary Table 6: Downregulated Gene Ontology Biological Process Terms in PBMC of SIV-infected monkey with long-term ART as compared to PBMC of Acute SIV-infected monkeys.

Category	Term	Genes	FoldEnrichment	PValue	FDR
GOTERM_BP_DIRECT	GO:0140374~antiviral innate immune response	ZBP1, MX1, EIF2AK2, IFIT1, IFIT3, IFIT2, OASL, IFIH1, CXCL10, OAS1, OAS2, OAS3, DHX58	31.1648	7.83267E-15	1.88376E-12
GOTERM_BP_DIRECT	GO:0045087~innate immune response	CX3CR1, IFIT5, IFI6, ADAR, DDX60, OASL, IFIH1, HERC5, DHX58, GBP2, GBP1, GBP3, RSAD2, MX2, MX1, ISG15, PARP14, PML, ISG20, ZAP70, OAS1, IFI27, OAS2, OAS3, KLRF1	6.834385965	1.63574E-13	3.14717E-11
GOTERM_BP_DIRECT	GO:0070106~interleukin-27-mediated signaling pathway	OAS1, STAT1, OAS2, OAS3, MX1, OASL	116.868	5.3173E-10	8.52541E-08
GOTERM_BP_DIRECT	GO:0060337~type I interferon-mediated signaling pathway	IFIH1, IFI27, OAS1, STAT1, OAS2, STAT2, OAS3, OASL	24.93184	2.84363E-08	3.90796E-06
GOTERM_BP_DIRECT	GO:0070098~chemokine-mediated signaling pathway	CX3CR1, CXCL10, CCL5, CCR5, CCR3	11.13028571	0.00103724	0.039912989
GOTERM_BP_DIRECT	GO:0071346~cellular response to type II interferon	STAT1, CCL5, GBP2, GBP1, GBP3	9.988717949	0.001551835	0.051478108
GOTERM_BP_DIRECT	GO:1900246~positive regulation of RIG-I signaling pathway	DHX58, DDX60, OASL	42.49745455	0.0021296	0.062081058
GOTERM_BP_DIRECT	GO:0071347~cellular response to interleukin-1	CCL5, GBP2, GBP1, GBP3	11.33265455	0.005191222	0.134971768
GOTERM_BP_DIRECT	GO:0006954~inflammatory response	CX3CR1, CXCL10, AFAP1L2, CCL5, NKG7, SIGLEC1, CCR5, ITGAL, CCR3	3.246333333	0.006460967	0.155386262
GOTERM_BP_DIRECT	GO:0051897~positive regulation of phosphatidylinositol 3-kinase/protein kinase B signal transduction	CX3CR1, PDGFRB, GPX1, CCL5, SPRY2, PRR5L	4.583058824	0.009895911	0.226663475

P value: nominal p value, FDR: False Discovery Rate (Benjamini–Hochberg method)

Supplementary Table 7: Upregulated Gene Ontology Biological Process Terms in PBMC of SIV-infected monkey with long-term ART as compared to PBMC of uninfected monkeys.

Category	Term	Genes	FoldEnrichment	PValue	FDR
GOTERM_BP_DIRECT	GO:0051897~positive regulation of phosphatidylinositol 3-kinase/protein kinase B signal transduction	SESN2, MAZ, OSM, ARRB2, CBL, RGL2, PIK3R5, PPARD	6.965740388	0.000150336	0.040590604
GOTERM_BP_DIRECT	GO:0045893~positive regulation of DNA-templated transcription	CCDC17, NFIC, IRF2, KMT2B, MAZ, PIM2, ARID1A, PML, PPARD	4.069458858	0.001666804	0.192873081
GOTERM_BP_DIRECT	GO:0006914~autophagy	LIMK2, ATG2A, CLEC16A, PLEKHM1, ULK1	6.642587484	0.006731353	0.419415101
GOTERM_BP_DIRECT	GO:0046426~negative regulation of receptor signaling pathway via JAK-STAT	SOCS3, SOCS1, CISH	22.74109362	0.0074322	0.430005885
GOTERM_BP_DIRECT	GO:0019221~cytokine-mediated signaling pathway	STAT5B, SOCS3, SOCS1, CSF3R, CISH	5.281401524	0.014736547	0.750398574
GOTERM_BP_DIRECT	GO:1901223~negative regulation of non-canonical NF-kappaB signal transduction	NLRP12, RASSF2, HDAC7	15.46394366	0.015749106	0.750398574
GOTERM_BP_DIRECT	GO:0006338~chromatin remodeling	PHF2, CARM1, ARID1A, BRD4, HCFC1	4.027068662	0.035413903	0.996309963
GOTERM_BP_DIRECT	GO:0007259~cell surface receptor signaling pathway via JAK-STAT	STAT5B, SOCS1, TNFRSF1A	9.20472837	0.041529634	0.996309963

P value: nominal p value, FDR: False Discovery Rate (Benjamini–Hochberg method)

Supplementary Table 8: Downregulated Gene Ontology Biological Process Terms in PBMC of SIV-infected monkey with long-term ART as compared to PBMC of uninfected monkeys.

Category	Term	Genes	FoldEnrichment	PValue	FDR
GOTERM_BP_DIRECT	GO:0006412~translation	LOC704012, RPS17, RPL37A, RPS3, LOC698197, RPSA, FAU, UBA52	14.54996024	9.97856E-08	1.17747E-05
GOTERM_BP_DIRECT	GO:0000398~mRNA splicing, via spliceosome	IK, RNF113A, PPIE, HTATSF1	14.49712814	0.002379634	0.096046449
GOTERM_BP_DIRECT	GO:0000028~ribosomal small subunit assembly	LOC720748, LOC697065, RPSA	39.60822511	0.002441859	0.096046449
GOTERM_BP_DIRECT	GO:0002181~cytoplasmic translation	LOC720748, LOC697065, RPSA	20.79431818	0.008599205	0.253676541
GOTERM_BP_DIRECT	GO:0019941~modification-dependent protein catabolic process	UBB, UBA52	85.31002331	0.02250378	0.531089199
GOTERM_BP_DIRECT	GO:0032743~positive regulation of interleukin-2 production	STOML2, RPS3	36.96767677	0.051192642	1
GOTERM_BP_DIRECT	GO:0006310~DNA recombination	XRCC6, ERCC5	18.79712378	0.098260409	1

P value: nominal p value, FDR: False Discovery Rate (Benjamini–Hochberg method)

Supplementary Table 9: Upregulated Gene Ontology Biological Process Terms in PBMC of SIV-infected monkey with long-term ART as compared to PBMC of SIV-infected monkey with short-term ART monkeys.

Category	Term	Count	%	PValue	Genes	List Total	Pop Hits	Pop Total	FoldEnrichment	Bonferroni	Benjamini	FDR
GOTERM_BP_DIRECT	GO:0032731~positive regulation of interleukin-1 beta production	10	4.524886878	3.91275E-08	PYCARD, EGR1, NLRP12, NLRP3, TLR8, NLRP1, NOD2, MEFV, TLR4, MYD88	213	66	19478	13.85545597	6.90967E-05	9.92125E-06	9.63473E-06
GOTERM_BP_DIRECT	GO:0051092~positive regulation of NF-kappaB transcription factor activity	10	4.524886878	6.65929E-06	PYCARD, SPHK1, CARD9, NLRP3, TRIM25, NOD2, S100A9, TLR4, MYD88, TLR2	213	120	19478	7.620500782	0.011691461	0.000842792	0.000818453
GOTERM_BP_DIRECT	GO:0043123~positive regulation of canonical NF-kappaB signal transduction	12	5.429864253	4.96537E-05	PYCARD, PELI2, CARD9, TRIM25, TLR8, LTBR, NOD2, TLR4, LITAF, MYD88, WLS, TNFRSF1A	213	232	19478	4.729966003	0.083955791	0.004175642	0.004055054
GOTERM_BP_DIRECT	GO:0002224~toll-like receptor signaling pathway	4	1.809954751	0.003066612	TLR8, TLR5, TLR4, TLR2	213	27	19478	13.54755695	0.99559034	0.102181826	0.099230935
GOTERM_BP_DIRECT	GO:0030890~positive regulation of B cell proliferation	4	1.809954751	0.01450372	BCL6, TNFSF13, TLR4, TNFSF13B	213	47	19478	7.782639097	1	0.294408843	0.285906662
GOTERM_BP_DIRECT	GO:0042116~macrophage activation	3	1.357466063	0.023606101	CD93, SLC11A1, TLR4	213	22	19478	12.46991037	1	0.412756186	0.400836273
GOTERM_BP_DIRECT	GO:0060907~positive regulation of macrophage cytokine production	5	2.262443439	0.000144218	PYCARD, CARD9, NOD2, TLR4, MYD88	213	25	19478	18.28920188	0.224856395	0.009795715	0.009512827
GOTERM_BP_DIRECT	GO:0048661~positive regulation of smooth muscle cell proliferation	6	2.714932127	0.000298698	PTAFR, PTGS2, IL6R, TLR4, MYD88, EREG	213	54	19478	10.16066771	0.40996813	0.019537034	0.018972828
GOTERM_BP_DIRECT	GO:0006915~apoptotic process	18	8.14479638	0.000873367	NOTCH2, HIP1, DAPK1, PRKCD, MX1, IFI6, YBX3, ZFP36L1, TNFRSF1A, PYCARD, NLRP3, CD14, NLRP1, LTBR, S100A9, MYD88, TNFRSF21, TLR2	213	656	19478	2.509189282	0.786269716	0.040588592	0.039416441
GOTERM_BP_DIRECT	GO:0032755~positive regulation of interleukin-6 production	12	5.429864253	1.46789E-08	PYCARD, SIGLEC16, CARD9, PTAFR, TLR8, NOD2, IL6R, TLR4, MYD88, EREG, IL17RA, TLR2	213	102	19478	10.75835405	2.59225E-05	7.13906E-06	6.93289E-06
GOTERM_BP_DIRECT	GO:0050729~positive regulation of inflammatory response	12	5.429864253	3.93254E-08	PYCARD, CEBPB, NLRP12, NLRP3, NLRP1, MEFV, NEAT1, S100A9, TLR4, IL17RA, TLR2, TNFRSF1A	213	112	19478	9.79778672	6.94463E-05	9.92125E-06	9.63473E-06
GOTERM_BP_DIRECT	GO:0042742~defense response to bacterium	13	5.882352941	3.90989E-07	NOTCH2, CEBPB, SLC11A1, PRKCD, FPR2, NOD2, CFP, MPEG1, TNFRSF1A, NLRP1, S100A9, TLR4, MYD88	213	171	19478	6.952035802	0.000690248	8.63108E-05	8.38183E-05
GOTERM_BP_DIRECT	GO:0071222~cellular response to lipopolysaccharide	13	5.882352941	1.34327E-06	NOD2, PTGS2, TNFRSF1B, LITAF, FCAR, PYCARD, SBNO2, RARA, NLRP3, CD14, TLR5, TLR4, MYD88	213	192	19478	6.191656886	0.002369412	0.000237222	0.000230371
GOTERM_BP_DIRECT	GO:0032760~positive regulation of tumor necrosis factor production	10	4.524886878	3.77161E-06	PYCARD, FCGR2A, CARD9, PTAFR, CD14, NOD2, TLR4, FCGR2C, MYD88, TLR2	213	112	19478	8.164822267	0.006638535	0.000555055	0.000539025
GOTERM_BP_DIRECT	GO:0002221~pattern recognition receptor signaling pathway	5	2.262443439	2.9051E-05	PYCARD, NLRP3, NLRP1, NOD2, MEFV	213	17	19478	26.89588511	0.050010932	0.002565203	0.002491123

P value: nominal p value, FDR: False Discovery Rate (Benjamini–Hochberg method)

Supplementary Table 10: Downregulated Gene Ontology Biological Process Terms in PBMC of SIV-infected monkey with long-term ART as compared to PBMC of SIV-infected monkey with short-term ART monkeys.

Category	Term	Count	%	PValue	Genes	List Total	Pop Hits	Pop Total	FoldEnrichment	Bonferroni	Benjamini	FDR
GOTERM_BP_DIRECT	GO:0006334~nucleosome assembly	25	35.71428571	1.23954E-33	8970, 8356, 8357, 8351, 3018, 3014, 8968, 8349, 8344, 8366, 8341, 8342, 8364, 8347, 8348, 8345, 8346, 8340, 8360, 3009, 3008, 3007, 3006, 8339, 121504	68	161	19478	44.47844355	3.19803E-31	3.19803E-31	3.09886E-31
GOTERM_BP_DIRECT	GO:0002227~innate immune response in mucosa	14	20	9.10063E-24	8970, 8344, 8341, 8342, 8347, 8348, 8345, 8346, 440689, 8340, 3018, 85236, 8349, 8339	68	37	19478	108.3831479	2.34796E-21	1.17398E-21	1.13758E-21
GOTERM_BP_DIRECT	GO:0019731~antibacterial humoral response	15	21.42857143	4.39561E-21	8970, 8344, 8341, 8342, 8347, 8348, 8345, 8346, 440689, 8340, 3018, 3512, 85236, 8349, 8339	68	76	19478	56.53444272	1.13407E-18	3.78022E-19	3.663E-19
GOTERM_BP_DIRECT	GO:0031507~heterochromatin formation	13	18.57142857	6.0055E-18	8330, 8331, 8336, 723790, 8334, 8335, 3012, 317772, 3014, 8329, 8338, 85235, 8969	68	68	19478	54.76081315	1.54942E-15	3.87355E-16	3.75344E-16
GOTERM_BP_DIRECT	GO:0061844~antimicrobial humoral immune response mediated by antimicrobial peptide	14	20	8.50453E-16	8970, 8344, 8341, 8342, 8347, 8348, 8345, 8346, 440689, 8340, 3018, 85236, 8349, 8339	68	135	19478	29.70501089	2.2915E-13	4.38834E-14	4.25226E-14
GOTERM_BP_DIRECT	GO:0050830~defense response to Gram-positive bacterium	14	20	1.37546E-15	8970, 8344, 8341, 8342, 8347, 8348, 8345, 8346, 440689, 8340, 3018, 85236, 8349, 8339	68	140	19478	28.64411765	3.43725E-13	5.9145E-14	5.7311E-14
GOTERM_BP_DIRECT	GO:0006325~chromatin organization	14	20	3.14885E-12	8970, 8366, 7153, 8364, 9734, 8356, 8357, 8335, 3012, 8351, 8360, 3009, 8968, 121504	68	254	19478	15.78809634	8.12394E-10	1.11926E-10	1.08455E-10
GOTERM_BP_DIRECT	GO:0032200~telomere organization	8	11.42857143	3.47057E-12	8366, 8364, 8356, 8357, 8968, 121504, 8351, 8360	68	27	19478	84.87145969	8.95404E-10	1.11926E-10	1.08455E-10
GOTERM_BP_DIRECT	GO:0061644~protein localization to CENP-A containing chromatin	7	10	2.36686E-11	8970, 8366, 8364, 8335, 3012, 121504, 8360	68	18	19478	111.3937908	6.1065E-09	6.785E-10	6.57461E-10
GOTERM_BP_DIRECT	GO:0045910~negative regulation of DNA recombination	4	5.714285714	2.55554E-05	3009, 3008, 3007, 3006	68	17	19478	67.39792388	0.00657169	0.000659329	0.000638885
GOTERM_BP_DIRECT	GO:0040029~epigenetic regulation of gene expression	5	7.142857143	3.55239E-05	9734, 8356, 8357, 8968, 8351	68	54	19478	26.52233115	0.009123449	0.000833196	0.000807361
GOTERM_BP_DIRECT	GO:0045653~negative regulation of megakaryocyte differentiation	4	5.714285714	4.25275E-05	8366, 8364, 121504, 8360	68	20	19478	57.28823529	0.010912344	0.000914341	0.000885989
GOTERM_BP_DIRECT	GO:0030261~chromosome condensation	4	5.714285714	6.55806E-05	3009, 3008, 3007, 3006	68	23	19478	49.81585678	0.016778001	0.001301522	0.001261165
GOTERM_BP_DIRECT	GO:0006959~humoral immune response	3	4.285714286	0.019891938	3512, 931, 1880	68	63	19478	13.64005602	0.994393626	0.366580002	0.35521318
GOTERM_BP_DIRECT	GO:0030183~B cell differentiation	3	4.285714286	0.024870534	9734, 974, 931	68	71	19478	12.1031483	0.998493156	0.427773186	0.414508901
GOTERM_BP_DIRECT	GO:0010467~gene expression	4	5.714285714	0.028880791	8356, 8357, 8968, 8351	68	193	19478	5.936604694	0.999479625	0.465702749	0.451262353
GOTERM_BP_DIRECT	GO:0007059~chromosome segregation	3	4.285714286	0.047340109	7153, 4288, 1063	68	101	19478	8.508153757	0.999996319	0.718455778	0.696178079

P value: nominal p value, FDR: False Discovery Rate (Benjamini–Hochberg method)
